# Nitrogen saturation drives shifts in response patterns of non-structural carbohydrate pools in a meadow steppe after ceasing nitrogen addition

**DOI:** 10.3389/fpls.2026.1792060

**Published:** 2026-03-25

**Authors:** Jie Yu, Shuai Wu, Jiaqi Ye, Yu Mo, Yu Zhao, Jing Zhang, Jiaxin Li, Yilin Zhang, Xunwen Wu, Liangchao Jiang, Guojiao Yang, Xiaotao Lü, Haiyang Zhang, Xingguo Han, Zhenghai Li, Yajing Bao

**Affiliations:** 1 College of Environment and Resources, Dalian Minzu University, Dalian, China; 2 State Key Laboratory of Vegetation and Environmental Change, Institute of Botany, Chinese Academy of Sciences, Beijing, China; 3 University of Chinese Academy of Sciences, Beijing, China; 4 School of Geography and Tourism, Qilu Normal University, Jinan, China; 5 Key Laboratory of Ecology and Resource Use of the Mongolian Plateau, Ministry of Education of China, School of Ecology and Environment, Inner Mongolia University, Hohhot, China; 6 College of Life Sciences, Hebei University, Baoding, China; 7 Key Laboratory of Agro-Forestry Environmental Processes and Ecological Regulation, School of Ecology and Environment, Hainan University, Haikou, China; 8 Erguna Forest Steppe Ecotone Research Station, CAS Key Laboratory of Forest Ecology and Management, Institute of Applied Ecology, Chinese Academy of Sciences, Shenyang, China

**Keywords:** historical nitrogen addition, nitrogen addition, nitrogen saturation threshold, non-structural carbohydrate pools, plant community

## Abstract

Non-structural carbohydrates (NSC) in grassland plants represent the outcome of carbon (C) allocation processes and serve as integrative indicators of plant C status and nutrient balance. However, their responses to different nitrogen (N) addition rates and to the cessation of long-term N addition remain unclear. In a temperate meadow steppe, we conducted a field experiment with six levels of N addition. We compared changes in the NSC pools and its components (soluble sugars, SS; starch, ST) at the community and functional-group levels. Community NSC and ST pools increased with increasing N addition rates, peaking at 20 g N m^-^² yr^-^¹, and subsequently declined after six years of continuous N addition. Three years after ceasing N addition, this single-peak response pattern changed, with no decline observed above the peak rate of 20 g N m^-^² yr^-^¹. NSC reserves were constrained by a saturation threshold of community N concentrations under N enrichment and N cessation scenarios. Before reaching this threshold, N addition increased community N concentrations and promoted the accumulation of the ST-dominated NSC pools. Above the threshold, the C-N coupling relationship weakened, and excessive N addition had negative effects on NSC and ST pools. From N enrichment to N cessation, the saturation threshold of community N concentrations shifted from 20 to 50 g N m^-2^ yr^-^¹, thus mitigating the adverse effects of high N addition on NSC pools. This study reveals the regulatory mechanisms of grassland C dynamics under the contrasting scenarios of N addition and N cessation and clarifies the differential responses of NSC reserves under N saturation constraints.

## 1 Introduction

Non-structural carbohydrates (NSC) are widely regarded as a core indicator in studies of plant carbon (C) allocation and C balance, reflecting the dynamic coupling between photosynthetic C supply and C consumption processes such as growth and maintenance (Wang et al., 2022). Soluble sugars (SS), as the most rapidly turning-over C form in metabolic networks, directly contribute to respiratory substrate supply, osmotic regulation, and the synthesis of defensive compounds (Xu et al., 2024). In contrast, starch (ST) typically stores C in a more stable form and can be hydrolyzed into SS when demand increases or supply is limited, thereby maintaining metabolic continuity (Huang et al., 2021; Martinez-Vilalta et al., 2016). Differences in turnover rates and mobilization pathways make NSC a key C buffer against source-sink fluctuations: when C supply is abundant, surplus assimilates are stored; when supply is limited, stored C is mobilized to sustain respiration and osmotic homeostasis, supporting physiological stability and subsequent growth (Dusenge et al., 2019; Hartmann and Trumbore, 2016). In grassland ecosystems, processes such as post-disturbance regrowth, overwinter survival, and spring green-up in herbaceous plants are often accompanied by phase-specific mismatches between photosynthetic supply and respiratory demand, making NSC levels and SS-ST composition closely linked to plant resilience and patterns of community renewal (Benot et al., 2018). Meanwhile, global climate change and accelerated human activities continue to alter the accumulation and allocation patterns of plant NSC pools and the ratios of their components (Tian et al., 2025; Du et al., 2020). Under stress conditions such as nitrogen (N) deposition, drought and extreme weather, NSC accumulation and mobilization are considered important pathways for enhancing stress tolerance and promoting recovery growth (El Omari, 2022; Signori-Müller et al., 2021). Therefore, characterizing the dynamic responses of NSC pools and their components from a comparative perspective of N enrichment versus N mitigation is crucial for revealing adaptive strategies of grassland plants and the mechanisms underlying ecosystem stability.

The Inner Mongolia grassland is an important ecological barrier in northern China and plays a key role in climate regulation, plant production, and C fixation (Guo et al., 2023). Its vegetation is dominated by grasses such as *Leymus chinensis* and *Stipa grandis*, and plant growth is limited by hydrothermal regime and soil nutrients (Bai et al., 2021; Su et al., 2020). Due to its unique hydrothermal regime and soil N limitation, the Inner Mongolia grassland is highly sensitive to N deposition (He et al., 2016; Li et al., 2022). N deposition not only alters soil chemical properties (Zhang and Han, 2012) but also affects bacterial communities and microbial biomass (Ning et al., 2022; Zhang and Han, 2012), thereby influencing the stability of grassland ecosystems. Thus, N deposition has become a key external driver regulating the source-sink balance between photosynthetic C input and growth C demand in meadow steppe and can reshape the accumulation patterns of plant NSC and its components. Existing studies indicated that the effects of N addition on NSC are jointly regulated by N input levels, organ types, and seasonal processes. Appropriate N addition (usually ≤ 10 g N m^-2^ yr^-1^) can enhance photosynthetic efficiency (Gong et al., 2022; Xie et al., 2022) and increase the uptake capacity of water and mineral nutrients in the rhizosphere (Zhu et al., 2021), thereby increasing SS and ST reserves in leaves and stems. N addition may also alter the allocation patterns of NSC among plant organs, and the magnitude of NSC fluctuations in roots is usually greater than that in leaves and stems (Zhu et al., 2021). A meta-analysis synthesizing 53 field experiments showed that, during the growing season, N addition has no overall significant effect on leaf and root NSC concentrations in woody plants; however, during the non-growing season, N addition reduces root NSC and ST concentrations by 13.8% and 39%, respectively, while increasing SS concentrations (Mou et al., 2024). Further studies reported that N addition significantly reduces NSC concentrations in foliage (Zhang et al., 2021), whereas NSC in above-ground woody tissues may respond positively under low to moderate N inputs (Li et al., 2019).

Within plants, NSC heterogeneity is primarily driven by interspecific differences, life-history strategies and functional types jointly determining the size and composition of NSC pools. For example, a global study spanning 308 woody species found that variations in root, stem, and twig NSC are mainly explained by species identity rather than habitat conditions or leaf habits, and that resource acquisitive species tend to maintain higher root reserves (Li and McDowell, 2026). Consistent with this pattern, a study in the Amazon rainforest showed that the slow-growing *Eschweilera coriacea* has whole-plant NSC stocks approximately 2.7 times those of the fast-growing *Bixa arborea*, largely due to higher ST storage in stems and roots (Signori-Müller et al., 2022). Moreover, stem ST concentrations are positively related with wood density, suggesting that life-history traits and structural characteristics constrain NSC storage capacity (Signori-Müller et al., 2022). Although much of this evidence comes from woody plants, the general principle that species differences and life-history strategies dominate NSC pool variations provides an important reference for understanding potential functional-group differentiation and C storage reallocation in the grassland community under N enrichment, and for predicting species- and functional-group-specific responses to N enrichment. At the ecosystem scale, N enrichment may not only alter within-individual C allocation but also amplify or offset individual-level NSC responses by shifting species dominance, thereby affecting community-level C storage and turnover. Long-term field experiments suggested that high N nitrogen inputs can increase the dominance of grasses while suppressing forbs, leading to declines in species richness and Shannon diversity (Zhang et al., 2022). Meanwhile, compared with woody plants, many herbaceous plants have weaker perennial storage organs and less lignified structures, potentially making community NSC pools more sensitive to changes in community composition (Zhou et al., 2025). Therefore, it is necessary to quantify the response patterns of NSC pools at the community and functional-group levels in the grassland ecosystem, and to elucidate how these differences are coupled with shifts in dominance hierarchy and community source-sink regulation.

In recent years, as global controls on N pollution have strengthened, atmospheric N deposition and anthropogenic N inputs have gradually declined (Hu et al., 2020). Whether ecosystems can recover their original structure and function after N inputs cease has become a frontier issue in global change ecology. Some studies have shown that the cessation of N addition can promote ecosystem recovery to some extent. In an 11-year multi-gradient N addition experiment in the Inner Mongolia grassland, the decreases in species richness and soil pH induced by N addition show signs of rapid recovery after ceasing N addition (Hao et al., 2018). In an alpine meadow of the Tibetan Plateau, soil N concentrations and pH recover rapidly after 15 years of continuous N addition (Yang et al., 2024b). However, high-intensity and long-term N addition often generate far-reaching legacy effects. A 30-year grassland N addition experiment showed that plant diversity has not recovered even 20 years after ceasing N addition (Isbell et al., 2013). Experiments in European upland heaths further demonstrated that changes in vegetation composition and declines in C pools induced by previous N addition and nitrogen-phosphorus co-additions persist even a decade after ceasing N addition (van Paassen et al., 2020). These findings indicated that the extent of ecosystem recovery after N cessation depends on the intensity and duration of previous N addition and on ecosystem type and is frequently accompanied by significant legacy effects. Importantly, such biotic and edaphic legacies are expected to extend to plant C allocation and storage (Lan et al., 2025; van Paassen et al., 2020), because NSC constitutes the primary C reserve that fuels maintenance, regrowth, and stress tolerance during recovery. However, apparent recovery in biodiversity or soil chemistry may not necessarily imply recovery of plant C reserves, and persistent legacy effects on NSC pools can mechanistically constrain the rebuilding of ecosystem C pools after N cessation. At present, most research has focused on community diversity and nutrient cycling, whereas the recovery dynamics of plant NSC pools remain poorly understood. Therefore, it is urgent to quantitatively assess the response and recovery trajectories of plant NSC after ceasing N addition using multi-gradient N addition-cessation experiments, to provide a scientific basis for evaluating and managing the recovery potential of grassland ecosystem C pools.

The purpose of this study was to analyze the effects of N addition and N cessation on plant NSC pools in a meadow steppe at the community and functional-group levels. In particular, under N-limited and N-saturated conditions, we analyzed the responses of NSC pools to N concentrations at the community and functional-group levels, thereby revealing the adaptive strategy underlying plant C allocation. Specifically, this study aims to address the following key questions: (1) How does N addition affect NSC, SS, and ST pools at the community and functional-group levels? What is the relationship between changes in these pools and N addition rates? (2) Do the responses of NSC, SS, and ST pools to historical N addition change at the community and functional-group levels after ceasing N addition? (3) Does the saturation threshold of N concentrations change at the community and functional-group levels after N cessation? If so, how does it affect NSC, SS, and ST pools at the community and functional-group levels?

## 2 Materials and method

### 2.1 Study site

The study was conducted in a temperate meadow steppe near the Erguna Forest-Steppe Ecotone Research Station of the Institute of Applied Ecology, Chinese Academy of Sciences, Inner Mongolia, China (50°10′46.1″N, 119°22′56.4″E). The field control experiment was established on natural grassland, and the site has been fenced to exclude livestock grazing since 2013. The long-term mean annual temperature is -2.45 °C, and the mean annual precipitation is 363 mm (based on 1957-2016) (Yang et al., 2023). The mean growing-season precipitation was 85 and 80 mm, and the mean growing-season temperature was 19 and 18 °C in 2019 and 2023, respectively (**Supplementary Figure 1**). The soil is classified as chernozem according to the Food and Agricultural Organization of the United Nations classification. The pH of the topsoil (0–10 cm) is 6.8-7.0. The nitrate-N and ammonium-N concentrations in the topsoil (0–5 cm) are 7.0-8.0 mg kg^-^¹ and 8.0-9.0 mg kg^-^¹, respectively. The background N-deposition rate was 1–2 g N m^-^² yr^-^¹ (Giese et al., 2013). The plant community was dominated by *L. chinensis*, *S. baicalensis*, and *Thermopsis lanceolata*.

### 2.2 Experimental design

An N addition experiment started in 2014 and ceased in 2021, following a completely randomized block design. For this study, we chose to add NH_4_NO_3_ as the N compounds. There were six levels of N addition rates (0, 2, 5, 10, 20, 50 g N m^-2^ yr^-1^), with eight replicates per treatment (Yang et al., 2023), and each plot was measured 10×10 m and separated by 1 m walkways. From 2014 to 2020, N fertilizer was applied annually at the beginning of growing season. NH_4_NO_3_ was thoroughly mixed with sand and then broadcast uniformly by hand to the N-addition plots. The sand was sieved through a 2-mm mesh, washed with water, and then oven-dried at 250 °C for 1 hour. To avoid potentially confounding effects, the same amount of sand (0.5 kg per plot) was added to all plots, including the controls.

### 2.3 Field sampling and measurement

Peak above-ground green biomass at the mid-August of 2019 (the sixth year of N addition) and 2023 (the third year of N cessation) was used to represent above-ground net primary productivity (ANPP) (Bai et al., 2004). Above-ground biomass was sampled annually using a 1×1 m quadrat. All samples were collected during daytime hours (approximately 08:00–17:00) and processed using identical sampling and pretreatment procedures to ensure consistency in sample handling and to minimize additional random variation caused by differences in plant phenological status associated with an extended sampling period. All living vascular plants were clipped and sorted to species. The quadrat was randomly placed in each plot without spatial overlap of quadrats among years and at least 50 cm inside the border of each plot to avoid edge effects. Plant samples were oven-dried at 65 °C for 48 hours and then weighed, ground with a ball mill (Retsch MM 400, Retsch GmbH & Co KG, Haan, Germany), and prepared plant samples. Specifically, the powdered plant samples (0.1000 g) were put into a 10 ml centrifuge tube, and 5 ml of 80% ethanol was added. The mixture was incubated in a water bath-shaker maintained at 80 °C for 30 min, and then centrifuged at 3500 rpm for 10 min. The pellets were extracted at least twice with 80% ethanol. Supernatants were retained, and pooled for assessment of SS. The ethanol-insoluble pellet was air-dried to evaporate the ethanol, and then the residue was used for ST extraction. To obtain ST, the residue was dispersed in 2 ml distilled water and heated for 15 min in a boiling water-bath. After cooling to room temperature, 2 ml of 9.2 M perchloric acid (HClO_4_) was added, and the mixture was shaken at room temperature for 15 min to allow for ST hydrolysis. Subsequently, 4 ml of distilled water was added to each sample, and then centrifuged at 4000 rpm for 10 min. The pellets were used for ST extraction one more time with 2 ml of 4.6 M HClO_4_. Supernatants were retained, combined, and topped up to 50 ml, and then used to determine starch concentrations. The concentrations of soluble sugars and starch were evaluated spectrophotometrically at 620 nm using the Anthrone colorimetry (Shimadzu UV-2600, Japan) (Wang et al., 2021). NSC concentrations were calculated as the sum of SS and ST concentrations. Not all species collected enough biomass for each quadrat, so a total of 1624 samples of 31 species were collected to determine NSC concentrations (**Supplementary Tables 1**, **2**). An elemental analyzer (EA-3000, Euro Vector, Italy) was used to evaluate plant N concentrations.

After clipping above-ground biomass and litter sampling at mid-August of 2019 and 2023, three soil cores (0–5 cm depth and 50 cm apart) in each quadrat were collected using a 7 cm diameter soil auger and mixed into one composite sample. Fresh soil samples were sieved through a 2-mm sieve to remove visible roots, plant residues, and stones, and taken to the laboratory for analysis of soil ammonium ( ${\text{NH}}_{4}^{+}$-N; mg kg^-1^) and nitrate ( ${\text{NO}}_{3}^{-}$-N; mg kg^-1^) concentrations and pH. To measure soil ${\text{NH}}_{4}^{+}$-N and ${\text{NO}}_{3}^{-}$-N concentrations, Fresh soil was extracted by 2 M KCl solution, 10 g of soil was extracted in 50 mL of 2M KCl solution, and then analyzed with a FLAstar 5000 Analyser (Foss Tecator). The soil ${\text{NH}}_{4}^{+}$-N and ${\text{NO}}_{3}^{-}$-N concentrations were expressed as mg kg^-1^ dry soil. Soil inorganic N concentration is the sum of soil ${\text{NH}}_{4}^{+}$-N and ${\text{NO}}_{3}^{-}$-N concentrations. Subsamples were air-dried and analysed soil pH using a pH meter (Thermo Fisher Scientific, America).

Plant functional groups were divided into three categories. The grassland is a meadow steppe dominated by *L. chinensis* and *S. baicalensis*. Grass plants have a high dominance, and legume plants have the particularity of N fixation of their rhizobia. Therefore, in this study, 31 species were divided into three functional groups: grasses (6 species in total), legumes (2 species in total), and forbs (non-grasses) (23 species in total) (Hector et al., 1999), and the specific classification is shown in **Supplementary Tables S1** and **S2**.

### 2.4 Statistical analysis

ANPP refers to the total biomass of all the species in a quadrat. That is:

(1) $$\text{ANPP}=\sum_{i=1}^{n}A_{i}$$

where $A_{i}$ represents the biomass of the species *i*, and *n* represents the number of species in a quadrat (**Equation 1**).

Relative biomass refers to the role and dominance of the species *i* in the community (or functional groups). That is:

(2) $$\text{Relative biomass}(\%)=\frac{A_{i}}{A}\times 100\%$$

where $A_{i}$ represents the above-ground biomass of the species *i*, and *A* represents ANPP (**Equation 2**).

Community (or functional group) N concentration is the overall mean of N concentrations across all species weighted by each species’ relative biomass in each quadrat (or functional groups). That is:

(3) $$\text{Community (or functional group) N concentration}=\sum_{i=1}^{n}B_{i}\times C_{i}$$

where Bi represents the relative biomass of species *i* in the community (or functional groups), Ci is the N concentration of the species, and *n* is the number of species included in the community (or functional groups) (**Equation 3**).

Community (or functional group) NSC/SS/ST pool is the total NSC/SS/ST accumulated per unit area at a certain time, which is equal to the total of the biomass of the species multiply its NSC/SS/ST concentration. That is:

(4) $$\text{Community (or functional group) NSC/SS/ST pool}=\sum_{i=1}^{n}A_{i}\times D_{i}$$

where $A_{i}$ represents the biomass of species *i* in the community (or functional groups), $D_{i}$ is the NSC/SS/ST concentration of the species *i*, and *n* is the number of species included in the community (or functional groups) (**Equation 4**).

We used the Quadratic-plus-plateau model to determine the response threshold of N concentrations in the plant community and grasses and corresponding N addition rates (Peng et al., 2020). According to the saturation threshold of N concentrations in the model, the quadratic-plus-plateau model can be described as follows:

(5) $$\text{y}={\text{ax}}^{2}+\text{bx}+\text{c if}(\text{x}<{\text{x}}_{\text{i}})$$

(6) $$\text{y}={\text{y}}_{\text{max}}\text{ if}(\text{x}\geq {\text{x}}_{\text{i}})$$

where x is N addition rates, x_i_ is the N addition rate when the dependent variable reaches the threshold point. a, b and c are regression coefficients of the quadratic (**Equation 5**), y_max_ is the maximum value of community and grass N concentrations (**Equation 6**). Based on the non-linear responses of N concentration to N addition or historical N addition, observations were classified into two phases: N-limited (below the N saturation concentration) and N-saturated (reaching or exceeding the N saturation concentration). We then analysed the effects of N concentrations in these phases on NSC and its components in the plant community and grasses (most species lost under high concentration of N addition rate).

Before conducting the analysis, all data were tested for normality using the Shapiro-Wilk and for homogeneity of variance using the Levene’s tests. The Multivariate Analysis of Variance (MANOVA) was applied to assess the effects of N addition rates, the sampling years, three types of pools and their interactions on NSC pools, its components and other variables. Best-fit models (quadratic or exponential decay) were performed based on Akaike Information Criterion (AIC) to examine the variations in the NSC pools and its components at the community and functional-group levels to N addition and historical N addition, using the ggplot2 and ggpmisc packages. The above statistical analysis was performed in R 4.4.2 (R Core Team 2024).

## 3 Results

### 3.1 Responses of the community- and functional-group-level NSC, SS and ST pools to N addition rates before and after ceasing N addition

**Figure 1:**
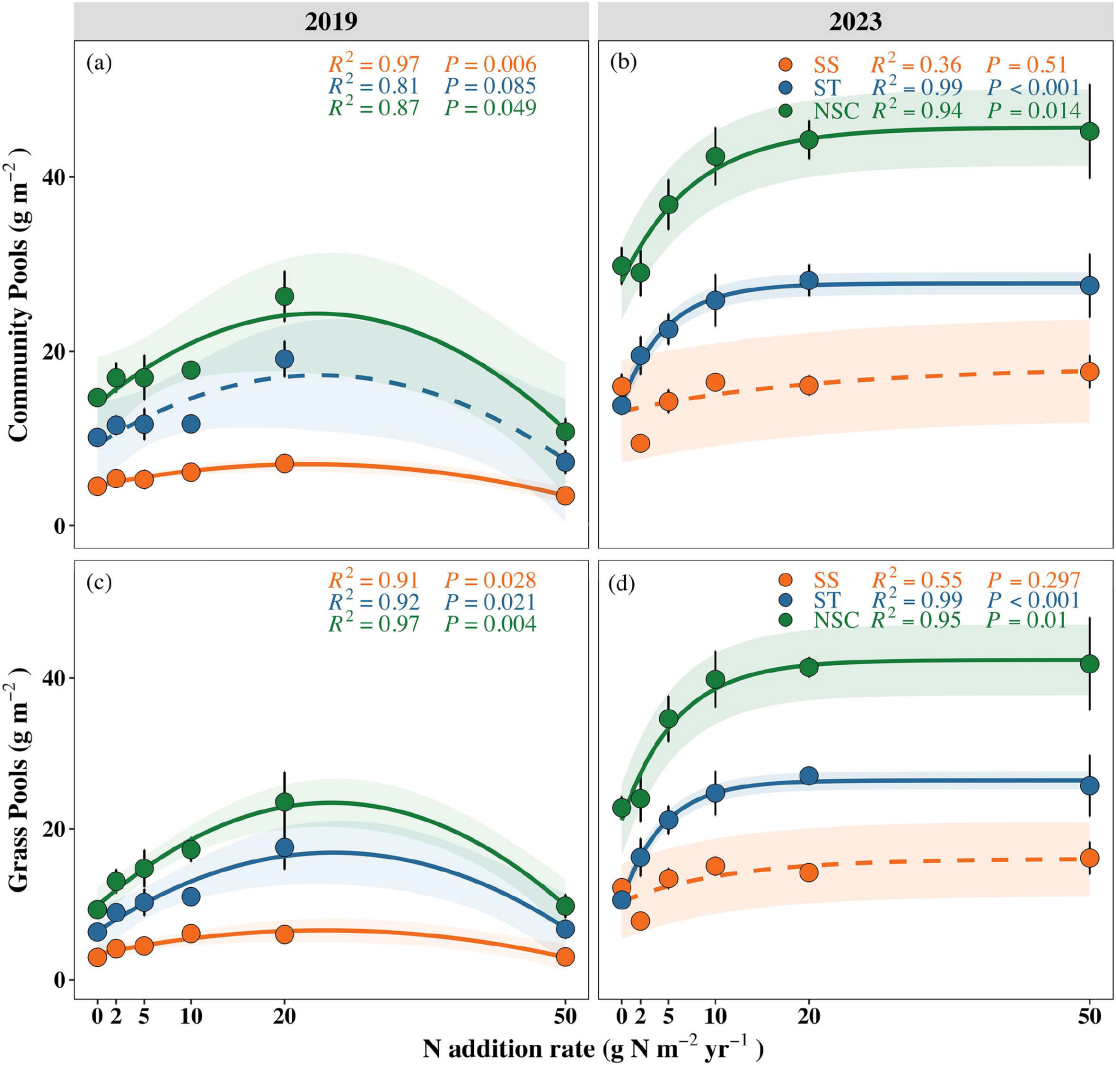
Responses of NSC, SS and ST pools in the community **(a, b)** and grasses **(c, d)** to N addition (2019) and historical N addition (2023). Orange, blue, and green represent SS, ST, and NSC pools, respectively. Solid lines indicate significant relationships (P < 0.05), whereas dashed lines indicate non-significant relationships. Data points represent means ± SE (n = 8).

In the six year of continuous N addition, community-level NSC and SS pools responded significantly to N addition (NSC: P = 0.049; SS: P = 0.006). Both pools exhibited an initial increase followed by a decline, peaking at 20 g N m^-^² yr^-^¹ (**Figure 1a**). In the third year after N cessation, community-level NSC and ST pools showed significant responses to historical N addition (NSC: P = 0.014; ST: P < 0.001). Both pools increased gradually under low and moderate historical N addition rates (0, 2, 5, 10, 20 g N m^-^² yr^-^¹), peaking at 20 g N m^-^² yr^-^¹, but did not decrease at higher historical N addition rates (20–50 g N m^-^² yr^-^¹) (**Figure 1b**). At the functional-group level, the trends of NSC, SS, and ST pools in grasses along both N addition and historical N addition were consistent with that of the plant community (**Table 1**; **Figures 1c, d**; NSC under N addition: P = 0.004; SS under N addition: P = 0.028; ST under N addition: P = 0.021; NSC under N cessation: P = 0.010; SS under N cessation: P = 0.297; ST under N cessation: P < 0.001), owing to the fact that grasses were the dominant functional group in the plant community (**Supplementary Figure 2**). In contrast, legumes and forbs showed no significant responses to either N addition or historical N addition (**Supplementary Table 3**; **Supplementary Figure 3**). Furthermore, ST pools were consistently higher than SS pools at the community and functional-group levels in both sampling years, indicating that plants tended to preferentially allocate surplus C to ST reserve (**Figure 1**).

**Table 1:** The MANOVA results for the effects of the sampling years (Y), N addition rates (N), categories (including three types of pools, C), and their interactions on pools at the community level and functional-group level in a meadow steppe.

	Community			Grass	
	*df*	*F*	*P*	*F*	*P*
N	5	15.147	<0.001	20.881	<0.001
C	2	225.978	<0.001	152.066	<0.001
Y	1	418.485	<0.001	315.668	<0.001
N*C	10	2.277	0.014	2.669	0.004
N*Y	5	10.407	<0.001	7.798	<0.001
C*Y	2	26.485	<0.001	20.442	<0.001
N*C*Y	10	1.386	0.187	0.876	0.556

The degrees of freedom (*df*) for the numerator are given.

### 3.2 The differential responses of the community- and functional-group-level NSC, SS and ST pools below and above N saturation threshold

**Figure 2:**
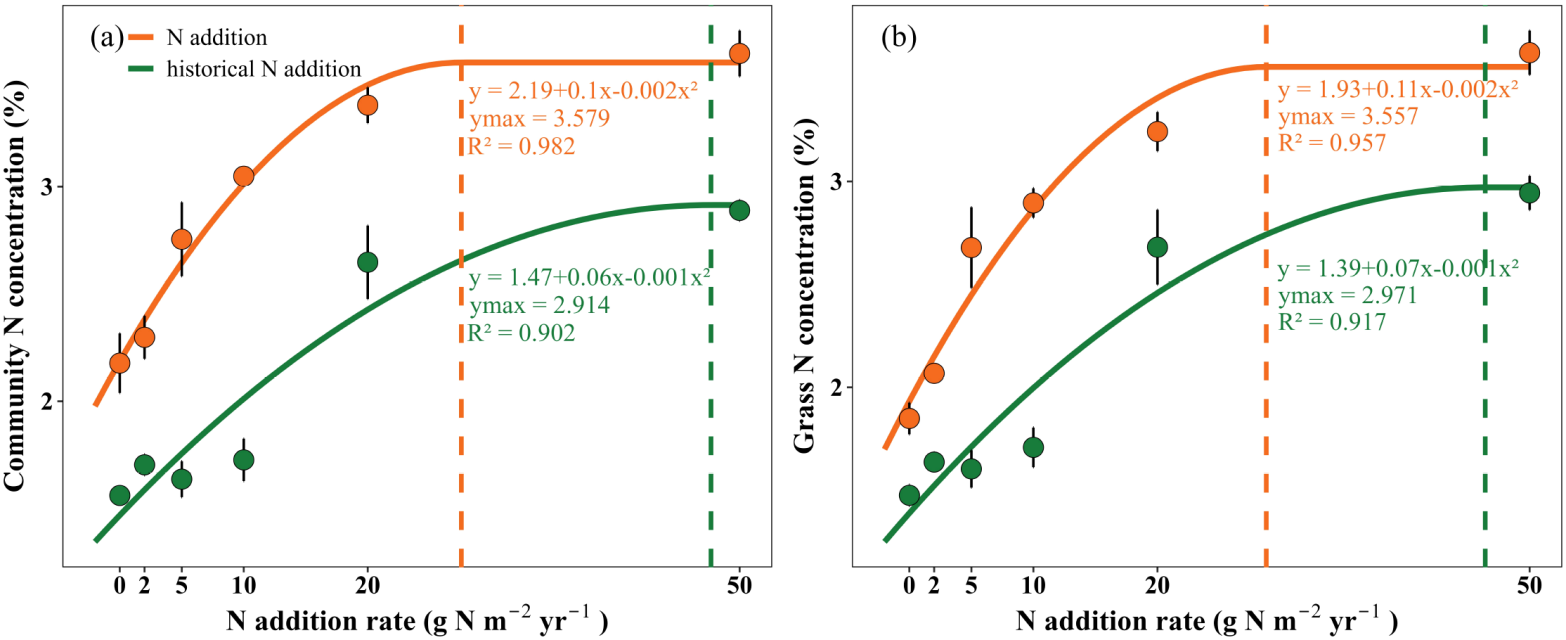
The Quadratic-plus-plateau model showed the non-linear responses of community **(a)** and grasses **(b)** N concentration to N addition and to historical N addition. Orange and green represent the measurements in 2019 (N addition) and 2023 (historical N addition), respectively. Fitted curves show the relationships between N concentrations and N addition rates for each year. Dashed vertical lines indicate the estimated N-saturation thresholds. Data points are means ± SE (n = 8).

The Quadratic-plus-plateau model analysis showed that the community N concentration reached a saturation threshold when the N addition rate exceeded 20 g N m^-^² yr^-^¹, and the community N saturation concentration was 3.579%. In the third year after N cessation, the saturation threshold shifted to nearly 50 g N m^-^² yr^-^¹, with an N saturation concentration of 2.914% (**Figure 2a**).

**Figure 3:**
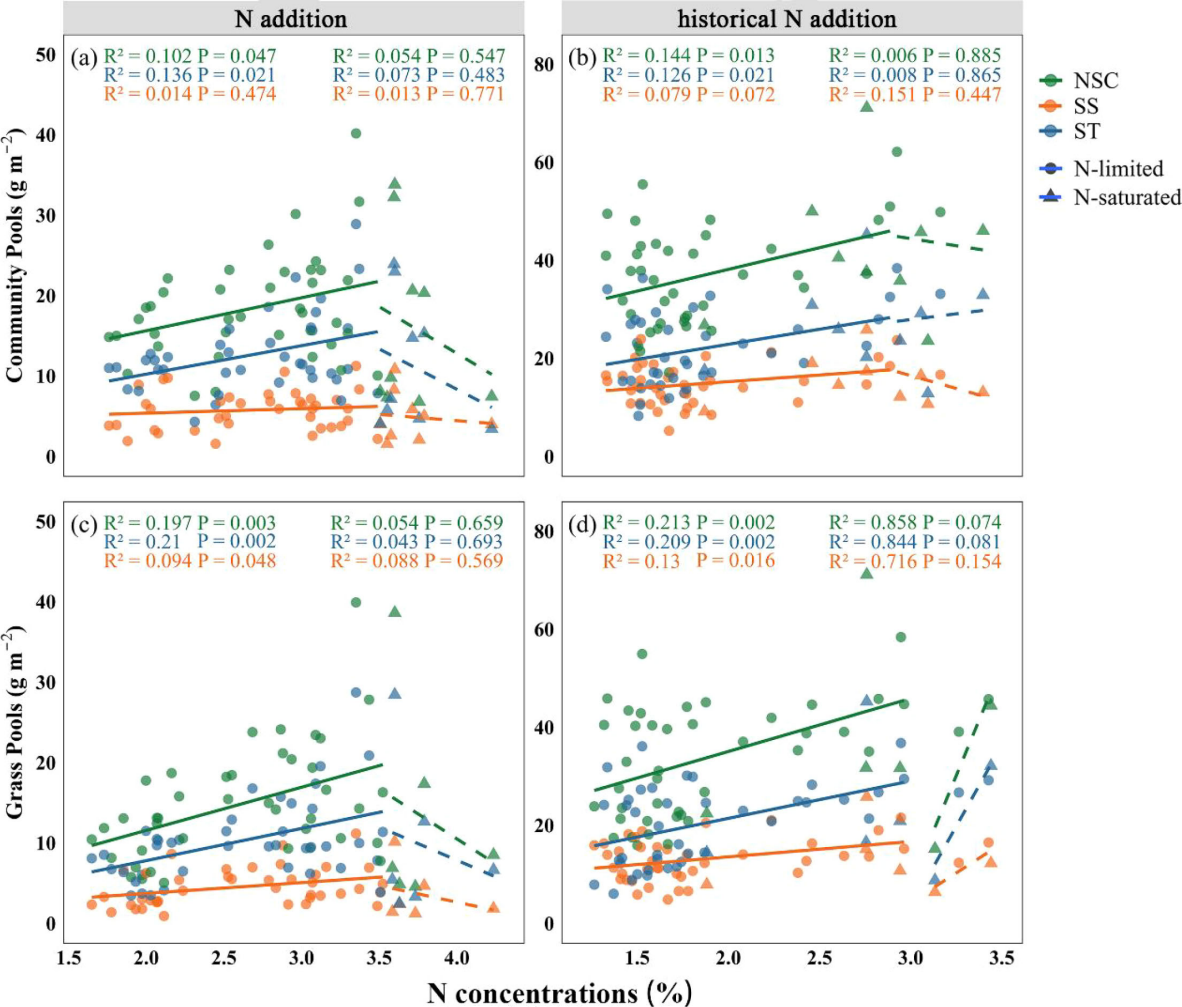
Relationships between NSC, SS and ST pools and N concentrations (N-limited and N-saturated) in 2019 (N addition) and 2023 (historical N addition). Orange, blue, and green denote SS, ST, and NSC pools, respectively. Circles and solid lines denote N-limited concentrations, while triangles and dashed lines represent N-saturated concentrations. Significance level: P < 0.05.

According to this classification, the plant community was divided into an N-limited phase and an N-saturated phase. MANOVA indicated under N-limited phase, community-level NSC and ST pools increased significantly with increasing community-level N concentrations (**Figures 3a, b**; NSC under N addition: P = 0.047; SS under N addition: P = 0.474; ST under N addition: P = 0.021; NSC under N cessation: P = 0.013; SS under N cessation: P = 0.072; ST under N cessation: P = 0.021). However, these relationships were no longer significant once community-level N concentrations reached or exceeded the saturation threshold (**Figures 3a, b**; NSC under N addition: P = 0.547; SS under N addition: P = 0.771; ST under N addition: P = 0.483; NSC under N cessation: P = 0.885; SS under N cessation: P = 0.447; ST under N cessation: P = 0.865). At the functional-group level, only grasses exhibited a non-linear response pattern consistent with that of the plant community, whereas legumes and forbs showed no significant changes under both phases (**Figure 2b–d**; NSC under N addition below the N saturation threshold: P = 0.003; SS under N addition below the N saturation threshold: P = 0.048; ST under N addition below the N saturation threshold: P = 0.002; NSC under N cessation below the N saturation threshold: P = 0.002; SS under N cessation below the N saturation threshold: P = 0.016; ST under N cessation below the N saturation threshold: P = 0.002; NSC under N addition above the N saturation threshold: P = 0.659; SS under N addition above the N saturation threshold: P = 0.569; ST under N addition above the N saturation threshold: P = 0.693; NSC under N cessation above the N saturation threshold: P = 0.074; SS under N cessation above the N saturation threshold: P = 0.154; ST under N cessation above the N saturation threshold: P = 0.081).

## 4 Discussion

### 4.1 N addition affected the community- and functional-group-level NSC, SS and ST pools

This study found that before 20 g N m^-^² yr^-^¹ treatment, the NSC and ST pools in the plant community and grasses increased significantly, showing a continuous upward trend. The study at the same study site found that long-term N addition increased ST concentrations in grasses but decreased those of forbs and legumes, and that changes in above-ground biomass were significantly and positively correlated with changes in NSC and N concentrations (Wang et al., 2021). Because N is the main limiting factor for vegetation growth in this area, plants tend to grow at a low speed under the condition of N deficiency (Liu et al., 2020). Moderate N addition not only stimulates growth but also promotes the accumulation of NSC pools. However, when N inputs exceeds plant growth or when other resources (e.g., light or water) become limiting, surplus photosynthates can no longer be utilized for plant growth and instead accumulate as NSC, leading to the observed NSC pools peaking at 20 g N m^-^² yr^-^¹. Similar studies have shown that exogenous N input can promote the rapid synthesis of available carbohydrates-SS in plants (Yang et al., 2024a) and accumulate in plant leaves (Martinez-Vilalta et al., 2016), and further promote the C fixation capacity of plants by increasing leaf area (Wang et al., 2019). Evidence has shown that low and moderate N addition rates can significantly increase the concentrations of NSC and its components (SS, ST) in leaves and branches, and promote the distribution and transport of photosynthates in plants, accelerating the overall growth process (Chen et al., 2025; Ning et al., 2018; Yang and Zhou, 2025).

Notably, in this study, the increases in NSC pools were primarily driven by ST accumulation. Previous studies have shown that ST plays a dominant role in regulating diel fluctuations of NSC pools and serves as an important C reserve in plants (Li et al., 2026). This pattern is closely linked to the short life cycle and seasonal growth strategy of grasses. Early in the growing season, grasses typically prioritize the accumulation of readily available SS to support rapid above-ground growth; later in the growing season or prior to dormancy, SS is progressively converted into ST, which serves as a long-term C reserve that provides C source for germination in the next growing season (Pouris et al., 2023; Purdy et al., 2015). Studies on bamboo species also support this pattern: ST depletion accompanies rapid branch and leaf development in spring, followed by renewed ST accumulation during the early stages of shoot formation (Song et al., 2016; Wang et al., 2016). Likewise, SS concentrations in the rhizomes of 2-3-year-old bamboo shoots exhibit a dynamic change of initial decline, subsequent rise, and eventual decline (Song et al., 2016). Therefore, the increases in ST-dominated NSC pools observed in our study likely reflects plant C allocation trade-offs during the growing season—a C reserve strategy that transitions from satisfying short-term demands for rapid growth to securing long-term survival and regeneration.

It should be noted that NSC concentrations may exhibit diurnal fluctuations, and sampling throughout the daytime may therefore introduce additional variance (Li et al., 2026; Tixier et al., 2018). However, because the sampling time showed no systematic bias among treatments, this influence is more likely to act as a random error rather than systematic bias. Consequently, it may affect the precision of absolute concentration estimates but is unlikely to alter the overall patterns of differences among treatments. Future studies could further reduce this uncertainty by standardizing sampling within a fixed morning time window.

### 4.2 The cessation of high N addition promoted the recovery of NSC, SS and ST pools

Our results showed that in the sixth year of N addition, community-level NSC and ST pools increased gradually with increasing N addition rates. However, when the N addition reached 50 g N m^-^² yr^-^¹, both pools declined significantly and even decreased below the unfertilized treatment. Previous studies have demonstrated that excessive N input can push plant growth toward the saturation threshold of productivity, eventually causing growth decline (Peng et al., 2020; Yang et al., 2023). A key mechanism underlying this response is that the high N addition rates inhibit photosynthesis and reduce C assimilation rates (Gong et al., 2022; Xie et al., 2022), thereby constraining the plant’s capacity for carbohydrate synthesis. Additionally, high N inputs can induce soil acidification (Guo et al., 2010) and ammonium toxicity (Namuhan et al., 2024). In our study, soil ammonium concentrations declined sharply under the high N addition treatment (50 g N m^-^² yr^-^¹) in the third year after N cessation (**Supplementary Figure 4**), which likely mitigated energy consumption, oxidative stress, and C-N metabolic imbalances induced by ammonium toxicity (Britto and Kronzucker, 2002; Xie et al., 2025). Photosynthates were no longer consumed in large quantities to cope with stress, thus enabling the re-accumulation and recovery of NSC, SS, and ST pools.

In contrast to N addition, NSC pools did not continue to decline under the high historical N treatment in the third year after N cessation. Instead, they stabilized at levels comparable to those under the 20 g N m^-^² yr^-^¹ treatment, demonstrating a clear recovery effect. This indicates that plants gradually restored a normal carbohydrate allocation strategy once N inputs were halted. As N was utilized and photosynthesis recovered, the negative effects of previous high N addition diminished, allowing plants to convert photosynthates more effectively into SS and ST. Even under conditions of the high historical N addition rate, NSC pools remained stable. Although soil inorganic N concentrations were still high (**Supplementary Figure 5**), plants appeared to shift their nutrient-utilized strategy from “overgrowth” to “resource conservation,” allocating more carbohydrates to NSC pools to guarantee energy supply and survival demand in the future growing season. In the third year after N cessation, photosynthates were no longer fully allocated to rapid above-ground growth but increasingly stored in roots and other long-lived organs. Evidence from previous studies further suggests that N-fixing microbes and mycorrhizal fungi gradually recover after N cessation (Fan et al., 2019; Högberg et al., 2011). We therefore speculate that plants may be able to acquire more bio-available N through renewed symbiotic associations, even though the N saturation concentration remained relatively high, thus reducing their dependence on exogenous N inputs. The functional restoration of microbial communities not only enhances plant nutrient acquisition capacity (Högberg et al., 2014) but also promotes carbohydrate reserves, thereby improving the stability of the NSC pools.

Differences in plant functional groups, life-history strategies, and growth forms may also influence NSC dynamics and their relationships with plant performance. In grassland ecosystems, annual herbs, perennial herbs, and semi-shrubs differ in C allocation strategies, reliance on stored C, tissue longevity, and growth phenology (Samraoui et al., 2025; Zhang et al., 2026). However, in this study, in contrast to grasses and the community-level patterns, legumes and forbs did not show significant or consistent responses after N cessation. This difference likely reflects the competitive hierarchy established under long-term N enrichment (Isbell et al., 2013). Grasses, with stronger resource acquisition and growth advantages under N addition, occupied dominant space and light resources, thereby suppressing legumes and forbs. In such a competition-driven context, interspecific competition may override the physiological responses of these functional groups to N cessation, masking potential changes in their NSC dynamics. Thus, functional group-specific responses and competitive interactions should be explicitly considered when interpreting community recovery processes.

While this study highlights the recovery effect of NSC pools after N cessation, we acknowledge that real-time variations in moisture and light were not systematically monitored during the recovery period. Given that these factors may strongly influence the rate and pattern of carbohydrate re-accumulation, their underlying regulatory mechanisms remain to be clarified. Future studies should use more detailed environmental monitoring methods to better elucidate the drivers of NSC recovery following nitrogen cessation.

### 4.3 N saturation threshold drove the adaptive changes of community- and functional-group-level NSC, SS and ST pools

Our results showed that NSC and ST pools in the plant community and grasses increased significantly with increasing N concentrations. However, once community N concentrations reached a saturation threshold, the positive relationship between community N concentrations and the increases in ST-dominated NSC pools disappeared; instead, NSC pools no longer increased and even declined with increasing community N concentrations. The result indicated that the C reserve strategy of plants underwent significant adaptive adjustment along the gradient from moderate to excessive N addition.

Moderate N supply promotes the increases in ST-dominated NSC pools, mainly due to the combined effects of the following aspects: first, N is an essential component of chlorophyll and key photosynthetic enzymes. Moderate N inputs enhance the concentrations and activities of enzymes such as Rubisco (Gong et al., 2022), improve stomatal and mesophyll conductance (Wang et al., 2019), and thereby increase photosynthetic efficiency (Gong et al., 2022), providing more substrates for carbohydrate synthesis (Peng et al., 2022). Second, when N supply is sufficient, plants reduce C investment in root growth (Yang et al., 2023) and N acquisition (Chen et al., 2020), thereby allowing more photosynthates to be stored as long-term reserves such as ST. Especially for grasses, surplus C is often stored in reserve organs under moderate N addition treatments to ensure subsequent growth and regeneration (Guo et al., 2022; Wang et al., 2019). Thirdly, moderate N addition improved plant nutritional status (Britz et al., 2023; Gao et al., 2020) and metabolic balance (Saiz-Fernández et al., 2017), making C allocation more tended to reserve accumulation rather than immediate consumption.

However, when the N supply exceeds the plant demand, these promotion mechanisms will change or be inhibited. Excessive N inputs may induce ammonium toxicity (Namuhan. et al., 2024, Van Den Berg et al., 2005) (**Supplementary Figure 4**), nutritional imbalances (Katzensteiner et al., 1992), and soil acidification (Yang et al., 2023) (**Supplementary Figure 6**), collectively reducing photosynthetic capacity. Under such conditions, Rubisco concentrations and activities decrease, and the supply of photosynthates becomes limited (Zhang et al., 2020). Meanwhile, plants need to consume more C skeleton and energy to assimilate and store excessive N (such as synthetic amino acids and proteins) under high N addition rates (Xin et al., 2019), resulting in a significant reduction in C sources available for ST accumulation. Furthermore, N saturation often triggers plant stress responses, shifting C-N allocation from reserves to structural repair and the maintenance of metabolic homeostasis (Zhang et al., 2025). Therefore, once community N concentration exceeds the saturation threshold, the previously increasing trend of ST-dominated NSC pools becomes weakened or even reversed.

This shift in C allocation pattern reflects the adaptive strategy of plants to excessive N: below N saturation, C reserve capacity is improved by accumulating ST and other reserve substances, and above N saturation, priority is given to maintaining physiological homeostasis to cope with environmental pressure to avoid metabolic imbalance and resources wastage.

## 5 Conclusions

With increasing N addition rates, the community-level NSC and ST pools showed a single peak response, peaking at the N addition rate of 20 g Nm^-2^ yr^-1^. In the third year after ceasing N addition, the single peak response trend of NSC and ST pools changed: there was no longer a downward trend above the N addition rate of 20 g Nm^-2^ yr^-1^. Whether N enrichment or N cessation, NSC reserves were constrained by the saturation threshold of community N concentrations. Before reaching the saturation, N addition promoted the increases in the community N concentrations, which was conducive to the accumulation of ST-dominated NSC pools. Above the saturation threshold, this C-N coupling relationship weakened, and excessive N addition began to have a negative impact on NSC and ST pools. After ceasing N addition, the saturation threshold of the community N concentration shifted from 20 g Nm^-2^ yr^-1^ during the N-addition period to 50 g Nm^-2^ yr^-1^, which mitigated the adverse effects of high N addition on NSC pools. This study revealed the differences in the responses of ST-dominated NSC pools under the constraint of the community N saturation threshold from N enrichment to the cessation of N addition. The results can provide a scientific basis for predicting grassland C dynamics and improving grassland management strategies under N deposition mitigation.

## Data Availability

The raw data supporting the conclusions of this article will be made available by the authors, without undue reservation.
