# Supplementary material for: Nitrogen saturation drives shifts in response patterns of non-structural carbohydrate pools in a meadow steppe after ceasing nitrogen addition
Source: Front Plant Sci. 2026 Mar 25;17:1792060. doi: 10.3389/fpls.2026.1792060 (PMC13057393; doi:10.3389/fpls.2026.1792060)
Supplement: Supplementary file 1 [file DataSheet1.pdf]

Supplementary Material

1 Supplementary Figures and Tables

1.1 Supplementary Figures

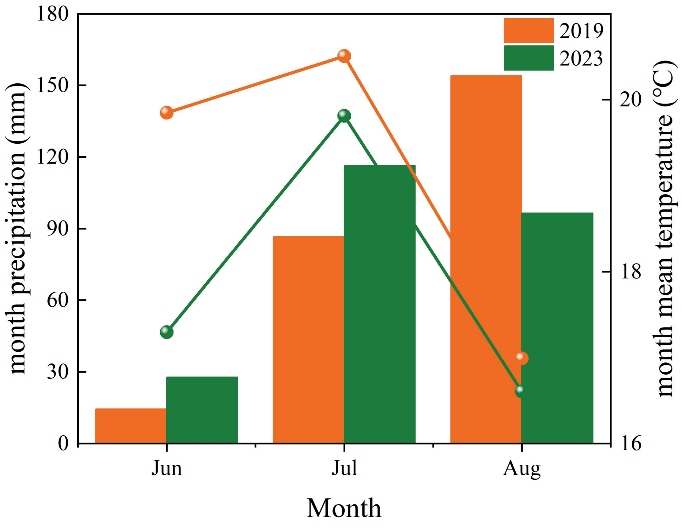

Supplementary Figure 1. The mean growing-season precipitation and mean growing-season temperature in 2019 (N addition) and 2023 (historical N addition). Orange and green represent year 2019 and 2023, respectively. Bars and lines with markers represent month precipitation and mean temperature, respectively.

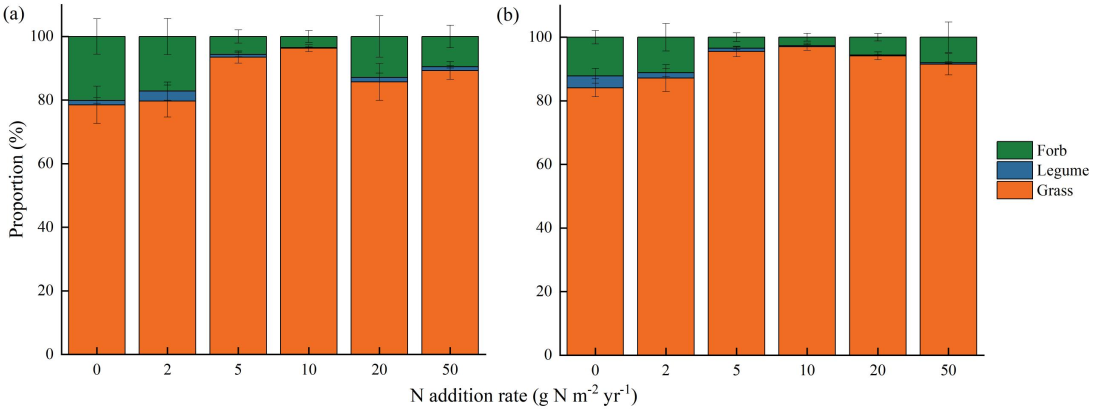

Supplementary Figure 2. The proportion of grasses, legumes and forbs in the community in 2019 (a) and 2023 (b). Orange, blue and green represent grasses, legumes and forbs, respectively. Data are means ± SE (n = 8).

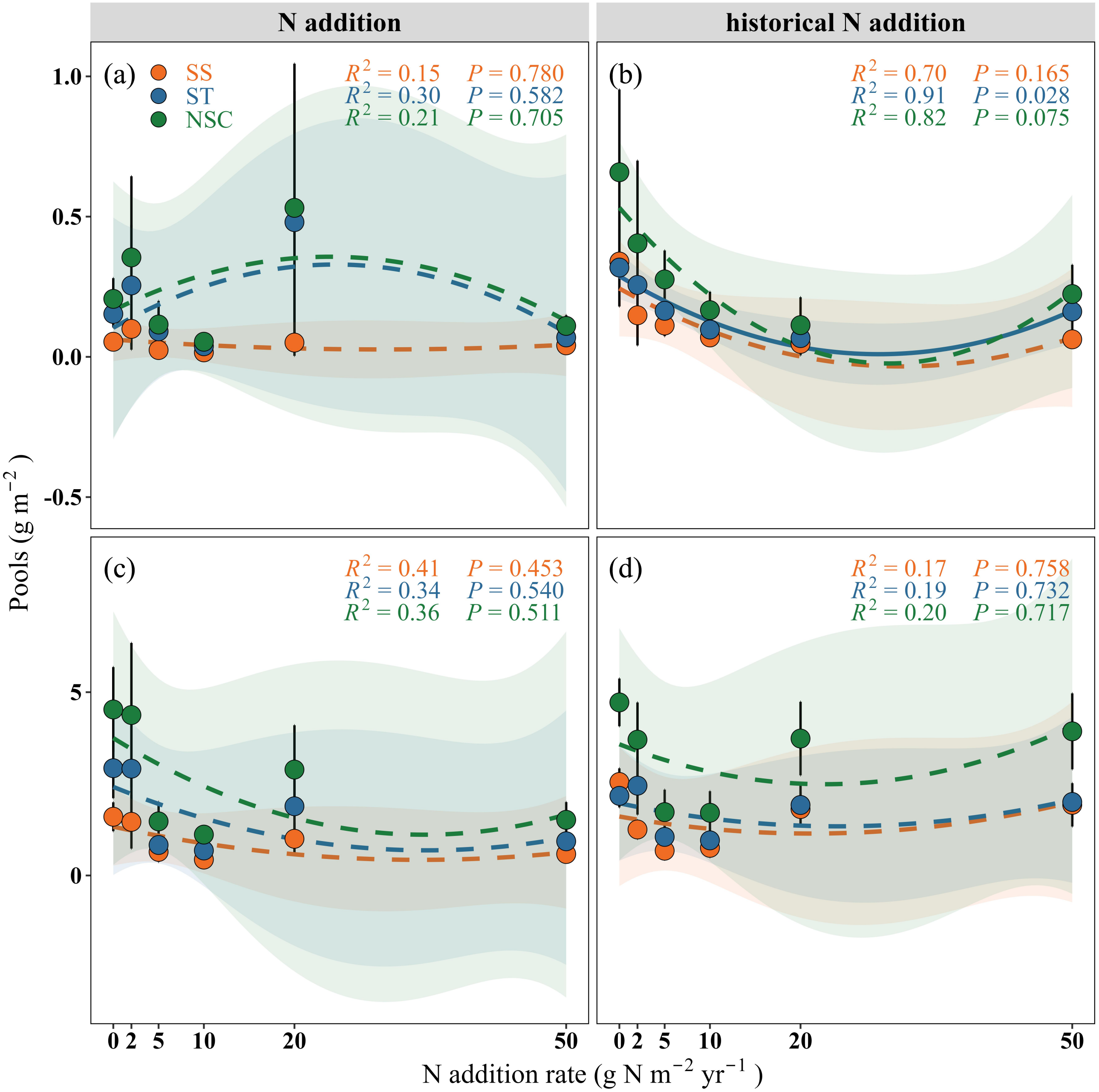

Supplementary Figure 3. Responses of NSC, SS and ST pools in the legumes (a, b) and forbs (c, d) to N addition (2019) and historical N addition (2023). Orange, blue, and green represent SS, ST, and NSC pools, respectively. Solid lines indicate significant relationships (P < 0.05), whereas dashed lines indicate non-significant relationships. Data points represent means ± SE (n = 8).

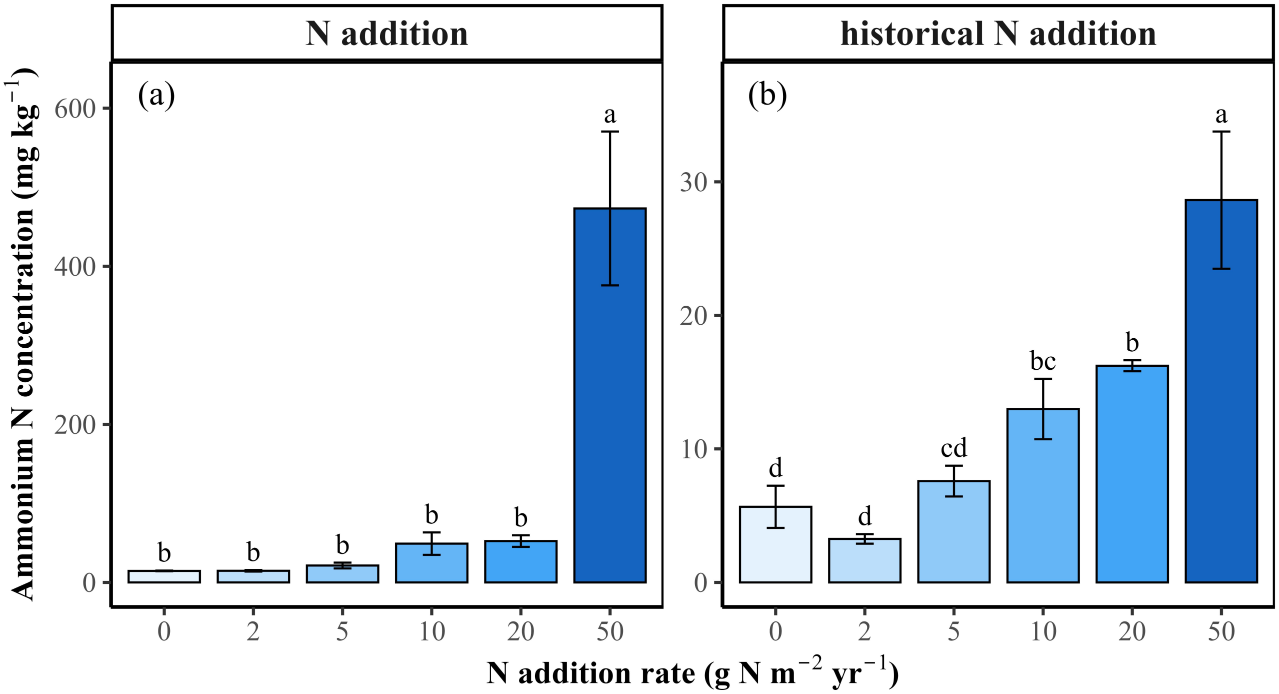

Supplementary Figure 4. Responses of soil ammonium N concentrations to N addition (a) and historical N addition (b). Different letters indicate significant difference at $P < 0.05$. Data are means ± SE (n = 8).

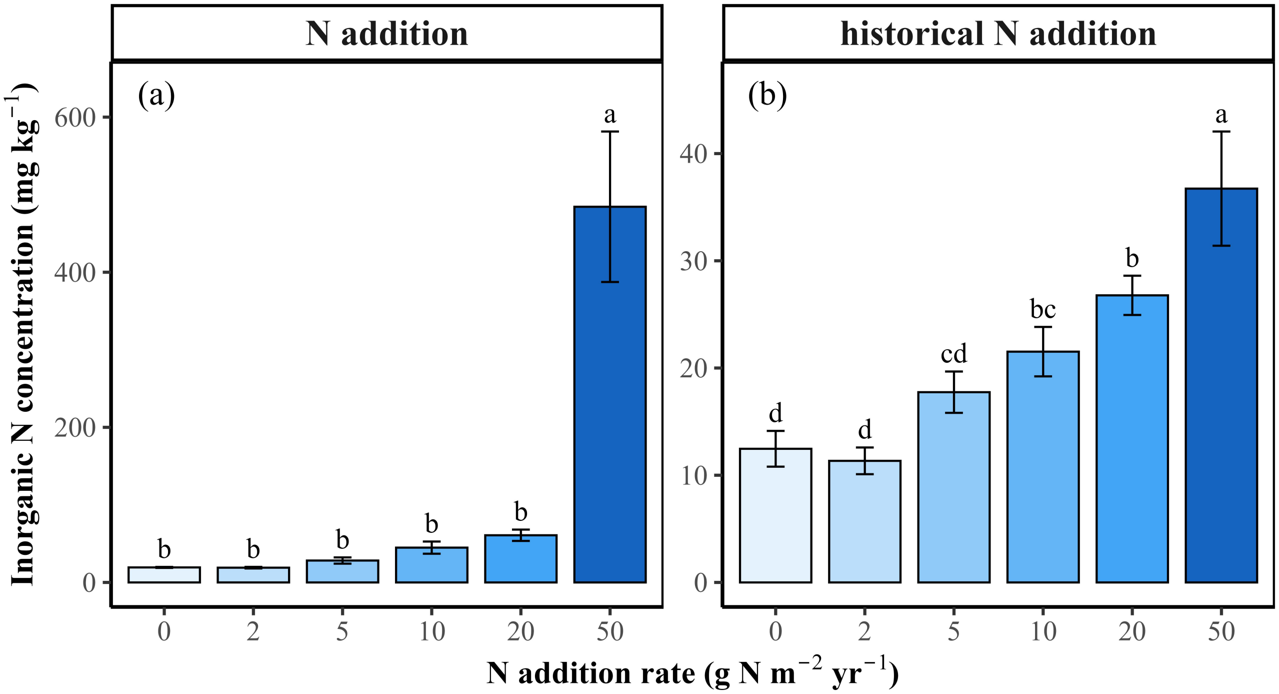

Supplementary Figure 5. Responses of soil inorganic N concentrations to N addition (a) and historical N addition (b). Different letters indicate significant difference at $P < 0.05$. Data are means ± SE (n = 8).

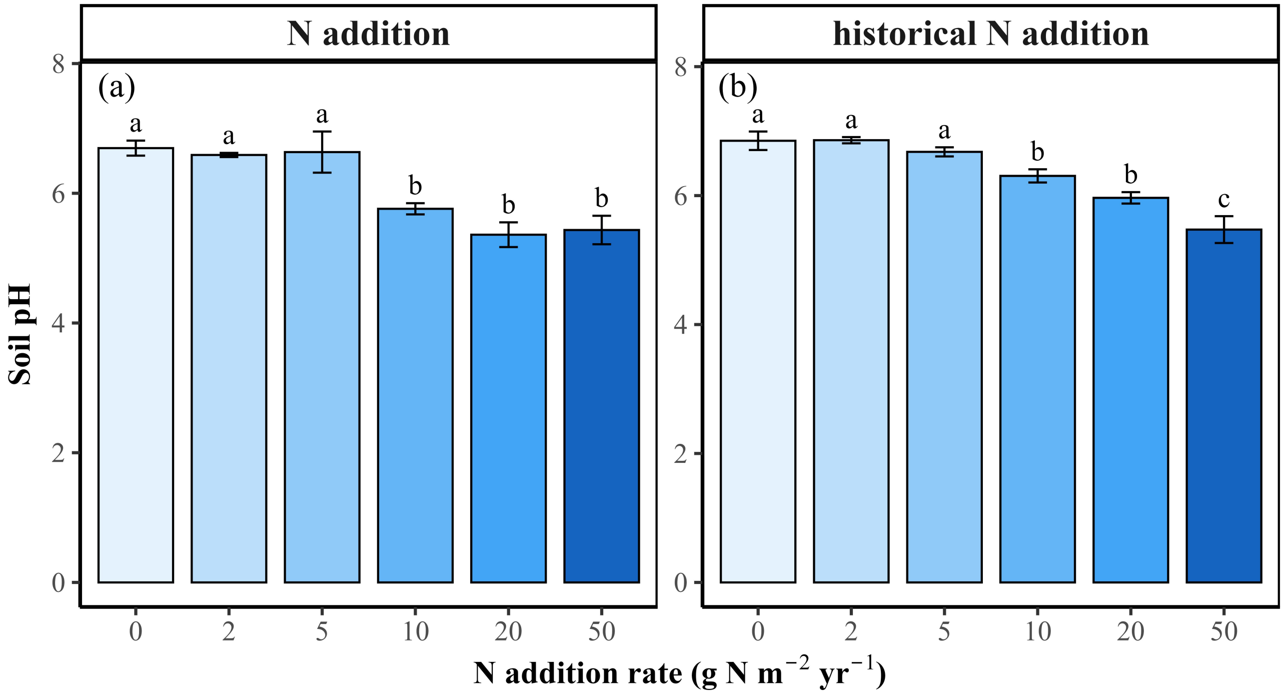

Supplementary Figure 6. Responses of soil pH to N addition (a) and historical N addition (b). Different letters indicate significant difference at P < 0.05. Data are means ± SE (n = 8).

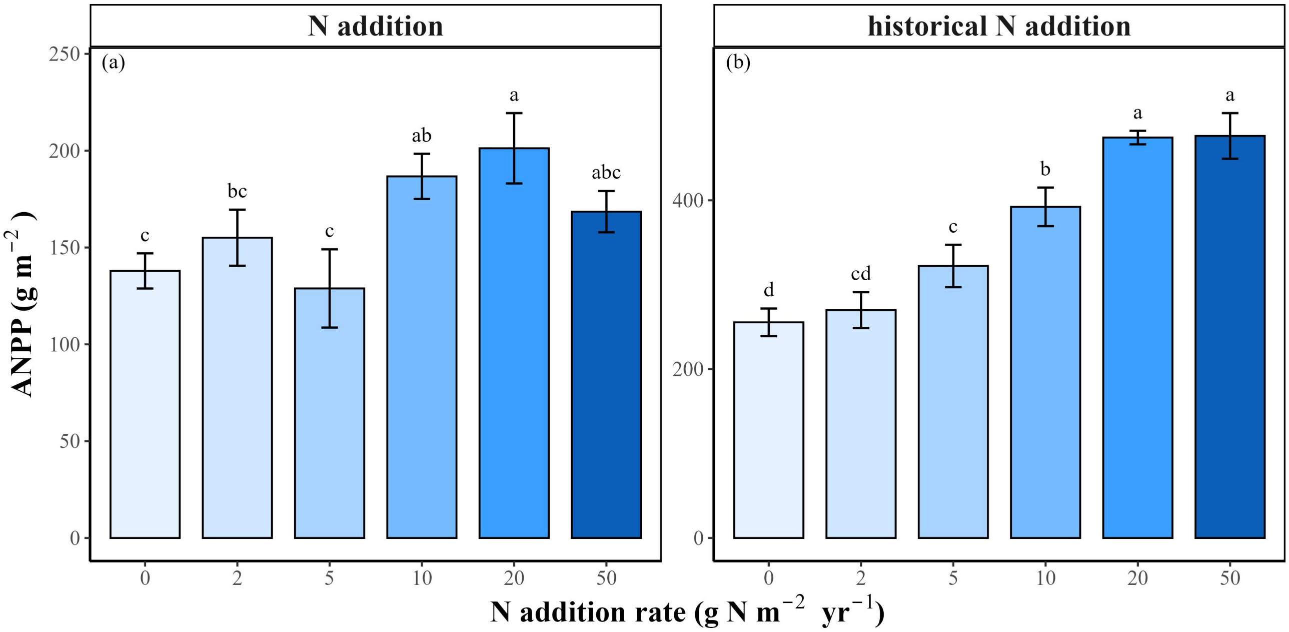

Supplementary Figure 7. Responses of ANPP to N addition (a) and historical N addition (b). Different letters indicate significant difference at P < 0.05. Data are means ± SE (n = 8).

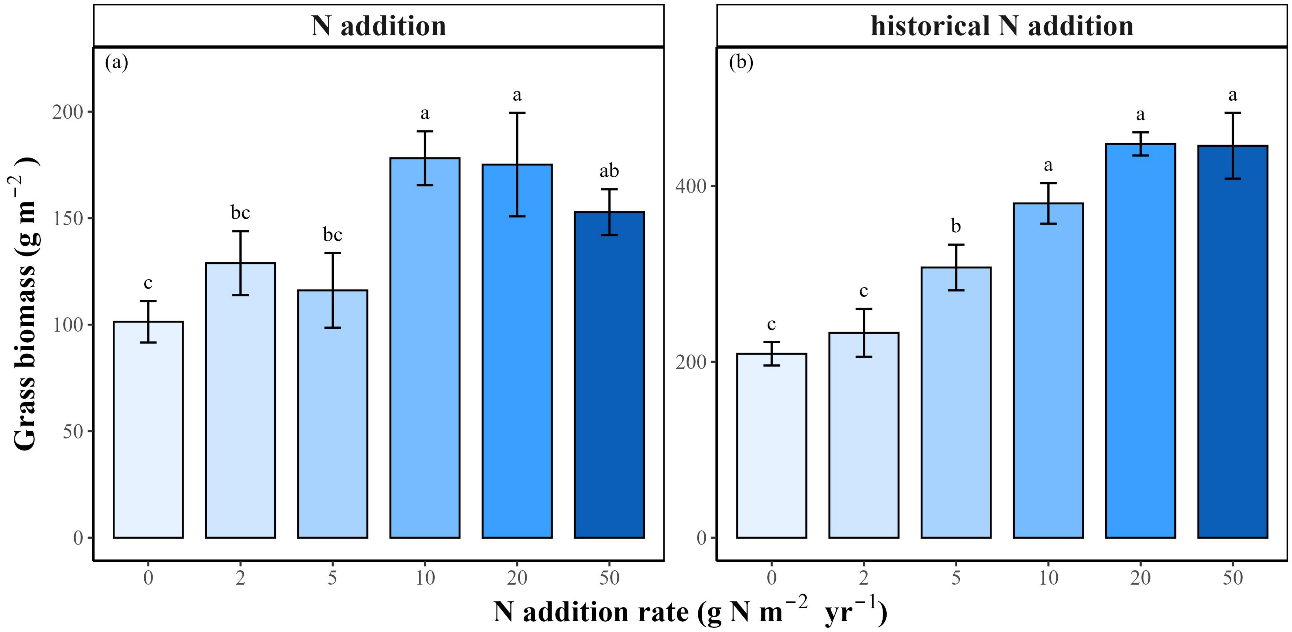

Supplementary Figure 8. Responses of grass biomass to N addition (a) and historical N addition (b). Different letters indicate significant difference at P < 0.05. Data are means ± SE (n = 8).

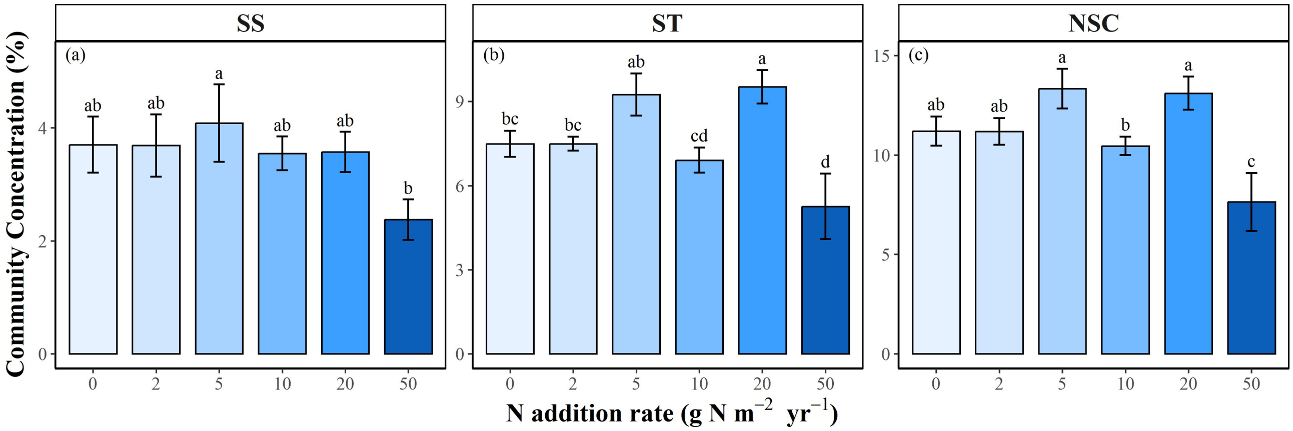

Supplementary Figure 9. Responses of Community SS (a), ST (b) and NSC (c) concentrations to N addition. Different letters indicate significant difference at P < 0.05. Data are means ± SE (n = 8).

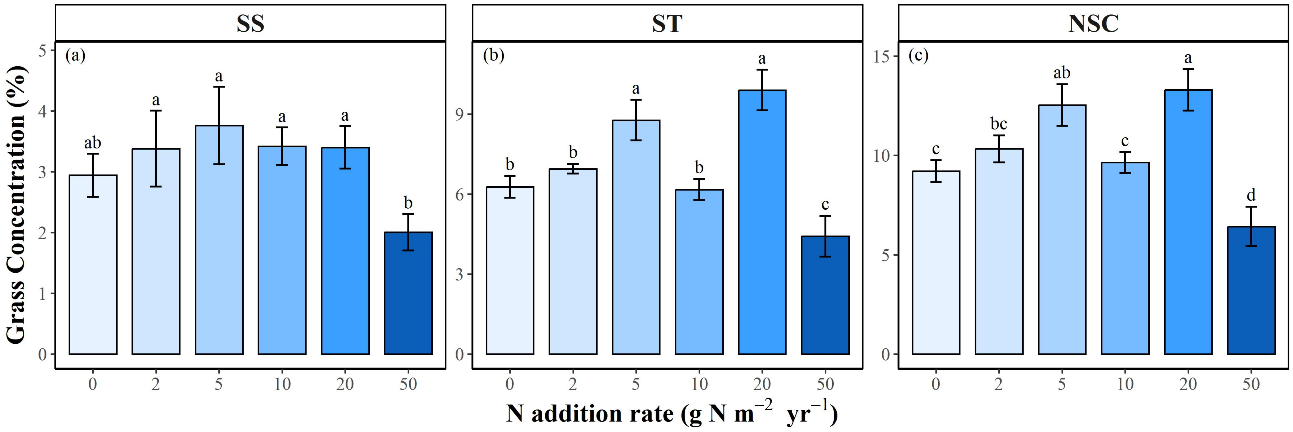

Supplementary Figure 10. Responses of grass SS (a), ST (b) and NSC (c) concentrations to N addition. Different letters indicate significant difference at P < 0.05. Data are means ± SE (n = 8).

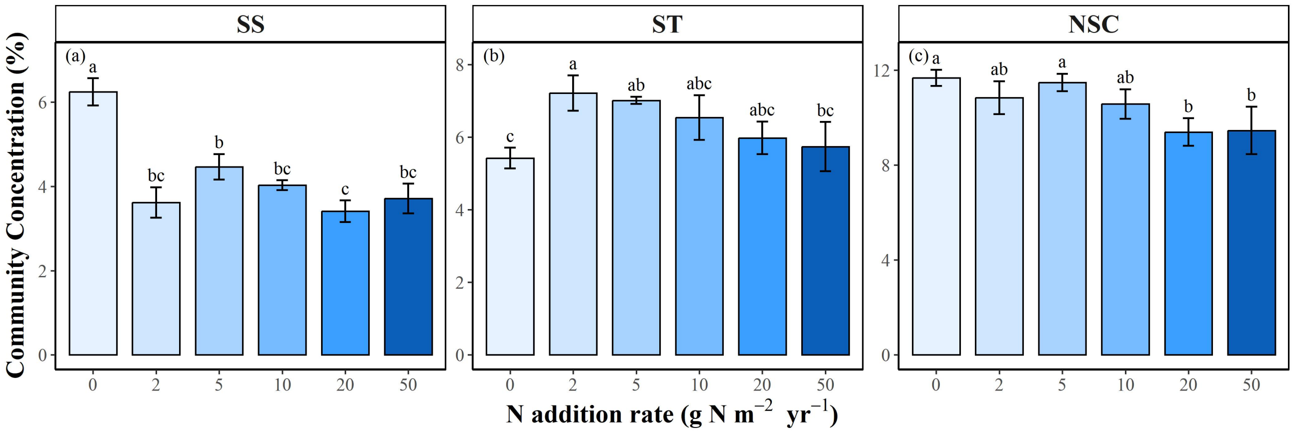

Supplementary Figure 11. Responses of Community SS (a), ST (b) and NSC (c) concentrations to historical N addition. Different letters indicate significant difference at P < 0.05. Data are means ± SE (n = 8).

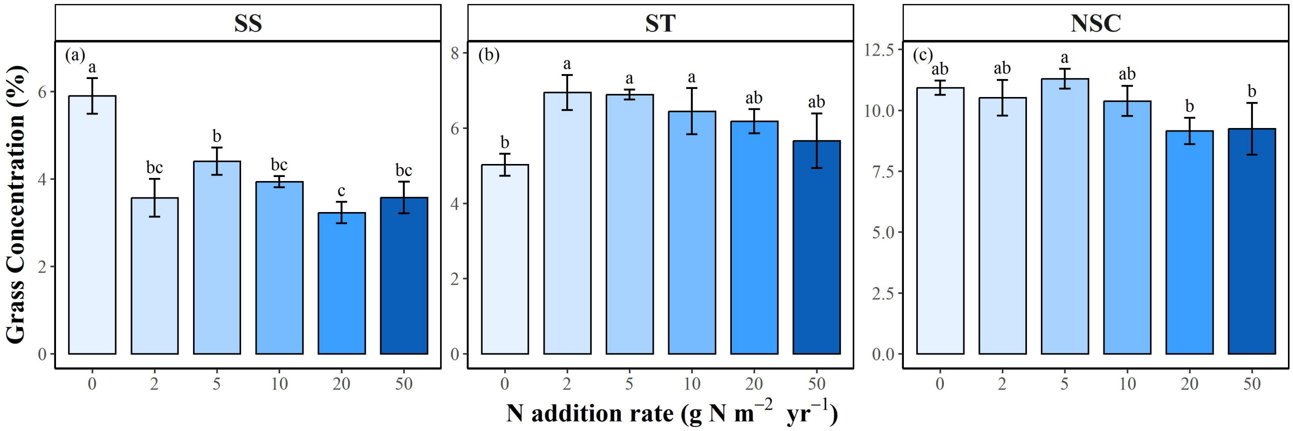

Supplementary Figure 12. Responses of grass SS (a), ST (b) and NSC (c) concentrations to historical N addition. Different letters indicate significant difference at P < 0.05. Data are means ± SE (n = 8).

1.2 Supplementary Tables

Supplementary Table 1. The family, relative biomass (year 2019) and other indices for NSC, SS and ST concentrations of all plant species recorded in our study. Species are listed in alphabetical order.

Species	Family	Relative biomass (%)	SS (mean ±SE)	ST (mean ±SE)	NSC (mean ±SE)	*Functional group*
Achnatherum sibiricum	Gramineae	0.27%	4.63±1.09	9.93±0.82	14.55±1.56	Grasses
Adenophora stricta	Campanulaceae	0.02%	5.03	7.30	12.33	Forbs

Allium ramosum	Liliaceae	0.00%	9.41	13.93	23.34	Forbs
Artemisia dracunculus	Compositae	1.43%	2.63±0.41	7.06±0.93	9.69±1.21	Forbs
Artemisia frigida	Compositae	0.36%	5.84±1.00	7.75±0.90	13.59±1.43	Forbs
Carex duriuscula	Cyperaceae	4.99%	4.14±0.45	7.69±0.63	11.83±0.77	Forbs
Chenopodium glaucum	Chenopodiaceae	0.18%	4.59±0.01	2.84±0.49	7.43±0.48	Forbs
Cleistogenes squarrosa	Gramineae	0.37%	4.10±0.73	8.22±0.67	12.32±1.16	Grasses
Cymbaria dahurica	Scrophulariaceae	1.42%	3.82±0.30	6.88±0.23	10.70±0.43	Forbs
Galium verum	Rubiaceae	0.12%	5.62±0.43	7.78±0.88	13.40±1.14	Forbs
Heteropappus altaicus	Compositae	0.65%	2.63	3.39	6.02	Forbs
Koeleria litvinowii	Gramineae	0.06%	6.67	7.34	14.01	Grasses
Leymus chinensis	Gramineae	83.84%	3.18±0.20	7.25±0.36	10.44±0.48	Grasses
Polygonum divaricatum	Polygonaceae	0.54%	3.95	10.71	14.66	Forbs
Potentilla bifurca	Rosaceae	0.90%	5.73±0.31	7.90±0.63	13.63±0.70	Forbs
Potentilla tanacetifolia	Rosaceae	0.32%	8.86	5.00	13.86	Forbs
Pulsatilla turczaninovii	Ranunculaceae	0.06%	4.19	8.77	12.96	Forbs
Serratula centauroides	Compositae	0.17%	6.00±0.36	8.41±0.52	14.41±0.68	Forbs
Stipa baicalensis	Gramineae	1.71%	2.23±0.30	6.47±0.74	8.70±0.78	Grasses
Thalictrum aquilegifolium	Ranunculaceae	1.12%	6.57±1.30	9.05±2.39	15.62±3.36	Forbs
Thermopsis lanceolata	Leguminosae	0.64%	3.57±0.46	8.46±1.01	12.04±1.32	Legumes

Supplementary Table 2. The family, relative biomass (year 2023) and other indices for NSC, SS and ST concentrations of all plant species recorded in our study. Species are listed in alphabetical order.

**Species**	**Family**	**Relative biomass (%)**	**SS (mean ±SE)**	**ST (mean ±SE)**	**NSC (mean ±SE)**	***Functional group***
Achnatherum sibiricum	Gramineae	0.21%	4.14	5.18	9.33	Grasses
Adenophora stricta	Campanulaceae	0.26%	3.89±0.40	5.57±0.35	9.44±0.45	Forbs
Allium ramosum	Liliaceae	0.06%	8.86±1.50	7.41±1.11	16.27±0.62	Forbs

Artemisia dracunculus	Compositae	0.11%	3.45±0.38	5.53±1.27	8.97±1.63	Forbs
Artemisia frigida	Compositae	0.15%	3.60±0.13	8.06±0.63	11.66±0.67	Forbs
Artemisia scoparia	Compositae	0.40%	3.87±0.65	5.48±1.03	9.34±0.93	Forbs
Artemisia tanacetifolia	Compositae	0.05%	4.42	5.33	9.75	Forbs
Bupleurum chinensis	Umbelliferae	0.01%	2.24±0.18	4.67±0.61	6.91±0.76	Forbs
Carex duriuscula	Cyperaceae	1.37%	4.60±0.34	5.85±0.32	10.45±0.43	Forbs
Chenopodium glaucum	Chenopodiaceae	0.01%	3.14±0.51	5.59±0.55	8.64±1.08	Forbs
Cleistogenes squarrosa	Gramineae	0.02%	6.50±1.01	6.11±1.62	12.61±2.50	Grasses
Cymbaria dahurica	Scrophulariaceae	0.09%	9.62±1.25	7.27±1.38	16.89±0.67	Forbs
Euphorbia esula	Euphorbiaceae	0.00%	6.67	11.99	18.66	Forbs
Galium verum	Rubiaceae	0.57%	6.45±0.30	5.92±0.49	12.36±0.44	Forbs
Heteropappus altaicus	Compositae	0.4%	2.92±0.46	6.19±0.87	9.11±0.83	Forbs
Koeleria litvinowii	Gramineae	0.10%	5.75±0.68	11.37±2.38	17.12±2.94	Grasses
Leymus chinensis	Gramineae	90.43%	4.10±0.18	6.15±0.21	10.28±0.28	Grasses
Linaria vulgaris	Scrophulariaceae	0.01%	7.38	9.44	16.82	Forbs
Poa annua	Gramineae	0.13%	3.41	14.43	17.84	Grasses
Potentilla bifurca	Rosaceae	2.11%	8.13±0.42	6.27±0.18	14.40±0.43	Forbs
Potentilla tanacetifolia	Rosaceae	0.01%	7.38±0.73	6.02±1.60	13.40±2.03	Forbs
Pulsatilla turczaninovii	Ranunculaceae	0.10%	10.18±0.93	7.65±1.88	17.83±2.75	Forbs
Sanguisorba officinalis	Rosaceae	0.07%	6.47	7.22	13.69	Forbs
Schizonepeta multifida	Labiatae	0.01%	2.33±0.22	4.88±0.61	7.20±0.72	Forbs
Sedum aizoon	Crassulaceae	0.00%	3.75	9.63	13.38	Forbs
Serratula centauroides	Compositae	0.20%	5.48±0.34	8.97±0.24	14.46±0.35	Forbs
Stipa baicalensis	Gramineae	0.03%	4.08	5.28	9.36	Grasses
Taraxacum mongolicum	Compositae	0.02%	3.80	4.74	8.54	Forbs
Thalictrum	Ranunculaceae	1.41%	6.50±0.27	7.60±0.59	14.09±0.77	Forbs

aquilegifolium						
Thermopsis lanceolata	Leguminosae	0.33%	4.25±0.17	6.31±0.26	10.56±0.19	Legumes
Vicia amoena	Leguminosae	0.61%	5.49±0.31	7.51±0.85	13.00±0.81	Legumes
